# Galiellalactone induces cell cycle arrest and apoptosis through the ATM/ATR pathway in prostate cancer cells

**DOI:** 10.18632/oncotarget.6606

**Published:** 2015-12-14

**Authors:** Víctor García, Maribel Lara-Chica, Irene Cantarero, Olov Sterner, Marco A. Calzado, Eduardo Muñoz

**Affiliations:** ^1^ Maimónides Biomedical Research Institute of Córdoba, Reina Sofía University Hospital, Department of Cell Biology, Physiology and Immunology, University of Córdoba, Córdoba, Spain; ^2^ Department of Science, Centre for Analysis and Synthesis, Lund University, Lund, Sweden

**Keywords:** galiellalactone, cancer, cell cycle, ATM/ATR, CHK1

## Abstract

Galiellalactone (GL) is a fungal metabolite that presents antitumor activities on prostate cancer *in vitro* and *in vivo*. In this study we show that GL induced cell cycle arrest in G_2_/M phase, caspase-dependent apoptosis and also affected the microtubule organization and migration ability in DU145 cells. GL did not induce double strand DNA break but activated the ATR and ATM-mediated DNA damage response (DDR) inducing CHK1, H2AX phosphorylation (fH2AX) and CDC25C downregulation. Inhibition of the ATM/ATR activation with caffeine reverted GL-induced G_2_/M cell cycle arrest, apoptosis and DNA damage measured by fH2AX. In contrast, UCN-01, a CHK1 inhibitor, prevented GL-induced cell cycle arrest but enhanced apoptosis in DU145 cells. Furthermore, we found that GL did not increase the levels of intracellular ROS, but the antioxidant N-acetylcysteine (NAC) completely prevented the effects of GL on fH2AX, G_2_/M cell cycle arrest and apoptosis. In contrast to NAC, other antioxidants such as ambroxol and EGCG did not interfere with the activity of GL on cell cycle. GL significantly suppressed DU145 xenograft growth *in vivo* and induced the expression of fH2AX in the tumors. These findings identify for the first time that GL activates DDR in prostate cancer.

## INTRODUCTION

Prostate cancer is the second most common diagnosed cancer in men worldwide and the first in developed countries. It has been estimated that 1.1 million new cases have occurred in 2012 [1]. Initially, prostate cancer depends on androgens for growth, and androgen deprivation therapy (ADT) is effective in the early stages of the disease. However, 18-24 months later, the majority of patients does not respond to ADT and develop a castration-resistant prostate cancer (CRPC), which is associated with a poor prognosis, and mean survival [2–5].

STAT3 belongs to the signal transducers and activators of transcription (STATs) family of transcription factors. STAT3 is activated in response to several growth factors and cytokines and is involved in numerous physiological processes such as inflammation, cell growth and differentiation. However, constitutive activation of STAT3 has been observed in many tumor types, including prostate cancer [6–9]. STAT3 regulates the expression of cell-cycle regulators, angiogenic factors and anti-apoptotic genes, promoting tumorigenesis [10].

Microtubules are essential components of the cytoskeleton and play a key role in division, growth and migration functions. Microtubule inhibitors (vinca alkaloids) or microtubule stabilizers (taxanes) have been among the most active chemotherapeutic drugs in treating human cancer [11]. Several studies have linked cytoplasmatic STAT3 with cytoskeletal structures. For example, cytoplasmatic STAT3 may modulate microtubule dynamics and cell migration through a direct interaction with stathmin protein that is a tubuling-binding protein involved in the control of microtubule assembly and dynamics. [12, 13]. Also, STAT3 inhibition decreases the migration of ovarian cancer cells and keratinocytes [14, 15]. Altogether, this suggests that inhibiting STAT3 activity may be an effective therapeutic strategy for cancer [16].

Galiellalactone (GL) is a fungal metabolite with potent antitumor and anti-inflammatory effects, isolated from *Galiella rufa* and it has also been produced synthetically [17]. GL is a direct inhibitor of STAT3 that prevents the binding of the activated STAT3 dimers to DNA binding sites without affecting tyrosine phosphorylation [18, 19]. GL is cytotoxic and induces apoptosis in androgen-insensitive prostate cancer cell lines and in prostate cancer stem cell-like cells. GL also inhibits tumor growth and early metastatic dissemination of prostate cancer in mice [20–22]. In addition, it has been demonstrated that GL inhibits NF-κB and TGF-β signaling, preventing the association of p65 with the importin α3 and inhibiting the binding of the activated Smad2/3 transcription factor to DNA, respectively [23, 24]. Also, GL improves experimental allergic asthma and it has an anti-thrombotic effect in murine models [25, 26].

In normal cells, the cell division cycle and apoptosis are tightly controlled, while cancer cells are characterized by deregulation in these processes [27, 28]. Checkpoints are the most important machinery involved in the control of the cell cycle. In response to genotoxic stress, DNA damage response (DDR) signaling pathway is activated, causing cell cycle arrest to allow the correction of the damage and to maintain genomic integrity. Checkpoints together with DNA repairing mechanisms and apoptosis are integrated in a circuitry that determines the ultimate response of a cell to DNA damage [29]. DNA damage is detected by MNR (MRE11, NBS1 and Rad50 proteins) and RPA (Human replication protein A) complexes act as sensors and recruit ataxia-telangiectasia mutated (ATM) and ataxia-telangiectasia and RAD3 related (ATR) to the site of the lesion, resulting in increased phosphorylation of histone H2AX (γH2AX), which is a marker of DNA damage. Activated ATM/ATR triggers phosphorylation of its downstream targets p53, CHK1 and CHK2, which in turn inhibit CDC25 phosphatases, preventing the activation of CDK1/Cyclin B and leading to G_2_/M arrest and initiation of DNA repair [30, 31]. Widely used drugs in cancer chemotherapy such as etoposide, cisplatin or doxorubicin are inducers of DNA damage pathway [32–34]. Therefore, the search for new effective drugs whose therapeutic target is ATM/ATR signaling may be a promising approach for CRPC treatment.

Natural products that induce cell cycle arrest and apoptosis have been an interesting source for the discovery of new therapeutic agents against cancer, including CRPC [35–37]. Our results provide first evidence that GL induces microtubules destabilization, DNA damage, G_2_/M cell cycle arrest and apoptosis through activation of the ATM/ATR pathway in the androgen-insensitive DU145 cells. Moreover, GL was able to induce the expression of γH2AX in DU145 xenograft tumors and therefore its antitumor effects may be due to the activation of DNA damage pathway by the same mechanism that occurs *in vitro*.

## RESULTS

### Galiellalactone induces cell cycle arrest and apoptosis in DU145 cells

**Figure 1:**
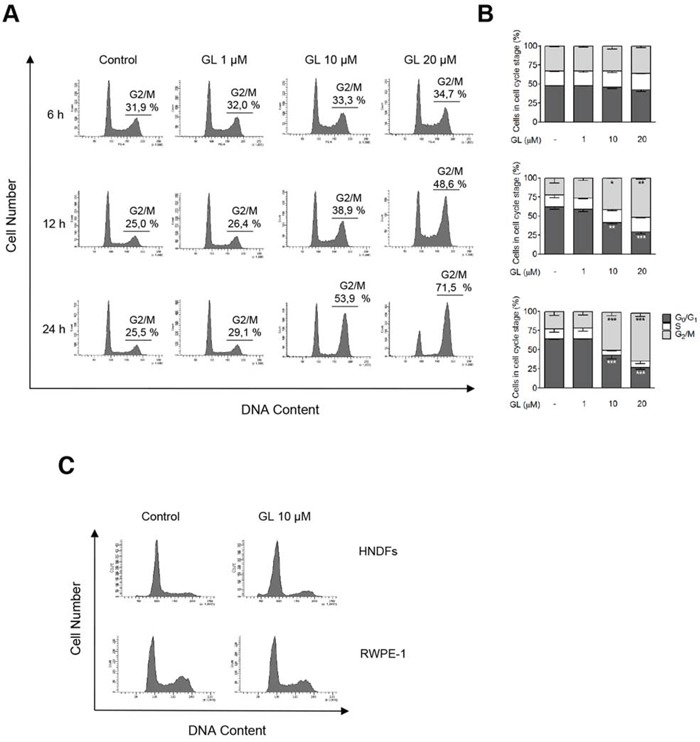
GL induces G_2_/M phase cell-cycle arrest **A.** DU145 cells were exposed to various doses of GL (1, 10 and 20 μM) during 6, 12 or 24 h and cell cycle was analyzed by PI staining and flow cytometry. Representative histograms are shown. **B.** Quantitation of percentages of the cells in each phase of the cell cycle. Data are the means of three independent experiments ± SD. *P<0.05; **P<0.01; ***P<0.001 compared with the control group. **C.** Effect of GL (24 h) on cell cycle in human normal dermal fibroblasts and RWPE-1 cells. Representative histograms are shown.

Since GL inhibits both STAT3 and NF-κB transcriptional activities, and both transcription factors participated in the progression of cell cycle in cancer cells [6, 38, 39], we were interested in studying the effect of GL on the cell cycle of prostate cancer cells. DU145 cells were treated with increasing concentrations of GL for 6, 12 and 24 h and the percentage of cells in the different phases of cell cycle identified by FACS analysis. We show in Figure 1A and 1B that GL induced a dose-dependent cell cycle arrest in the G_2_/M phase that was more evident after 24 h of treatment in DU145 cells. Similar results were obtained in other human cancer cells like Jurkat or SK-N-SH (data not shown), and human prostate cancer cell line PC3 (Supplementary Figure 1). The different p53 expression between the cell lines analyzed (p53 wild-type and null) indicated that GL induces G2/M phase cell cycle arrest independent of p53. In the same sense, PC3 cells (p53 null) transfected to express p53 wild-type showed analogous effects in response to GL (Supplementary Figure 1). In contrast, GL did not induce cell cycle arrest either in primary fibroblasts or in non-tumorigenic RWPE-1 cells that are derived from prostate epithelium (Figure 1C).

**Figure 2:**
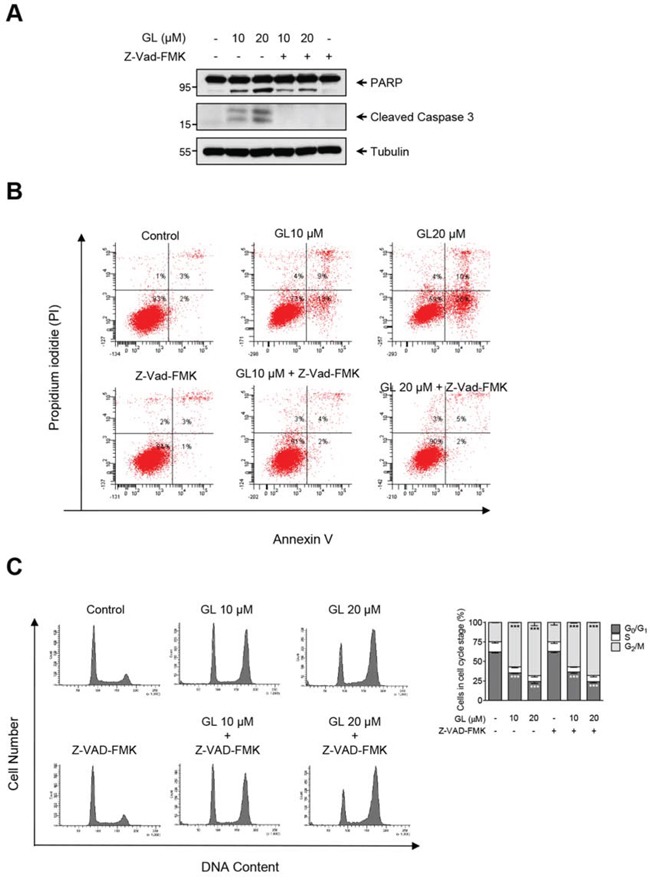
GL induction of cell-cycle arrest is mediated by a caspase-independent pathway **A.** DU145 cells were treated with GL in the absence or the presence or the pan-caspase inhibitor Z-VAD-FMK (40 μM) for 48 h and protein expression of PARP and cleavaged caspase-3 was analyzed by immunoblot. **B.** DU145 cells were treated as above for 48 h, stained with Annexin V and PI and analyzed by flow cytometry. Representative plots and percentages are shown. **C.** DU145 cells were treated as in A and cell cycle distribution was determined by flow cytometry. Quantitation of percentages of the cells in each phase of the cell cycle. Data are the means of three independent experiments ± SD. ***P<0.001 compared with the control group.

Previous reports have shown that GL induces apoptosis in DU145 cells through a caspase-3 dependent pathway [20]. Thus, we investigated whether cell cycle arrest paralleled with caspase-3 activation and apoptosis. DU145 cells were pre-incubated with the cell-permeant pan caspase inhibitor Z-Vad-FMK and treated with GL. We found that GL induced the activation and cleavage of caspase-3 that preceded the membrane translocation of phosphatidyl-serine measured by Anexin-V staining and both activities were completely inhibited in the presence of Z-Vad-FMK (Figures 2A and 2B). On the contrary, pan caspase inhibitor did not prevent GL-induced G_2_/M phase cell cycle arrest (Figure 2C). These results indicate that GL affects different signaling pathways in DU145 cells, leading to cell cycle arrest and apoptosis.

### Galiellalactone destabilizes microtubules and inhibits cell migration in DU145 cells

Actin and tubulins are abundant cytoskeletal proteins that support diverse cellular processes including cell cycle progression. To investigate the molecular and cellular mechanisms of GL effects on cell shape, we evaluated cell morphology using confocal microscopy, comparing the effects induced by cytochalasin D, a blocker of actin polymerization and elongation of actin, with those induced by nocodazole and docetaxel, two antineoplasic agents that interfere microtubules polymerization.

**Figure 3:**
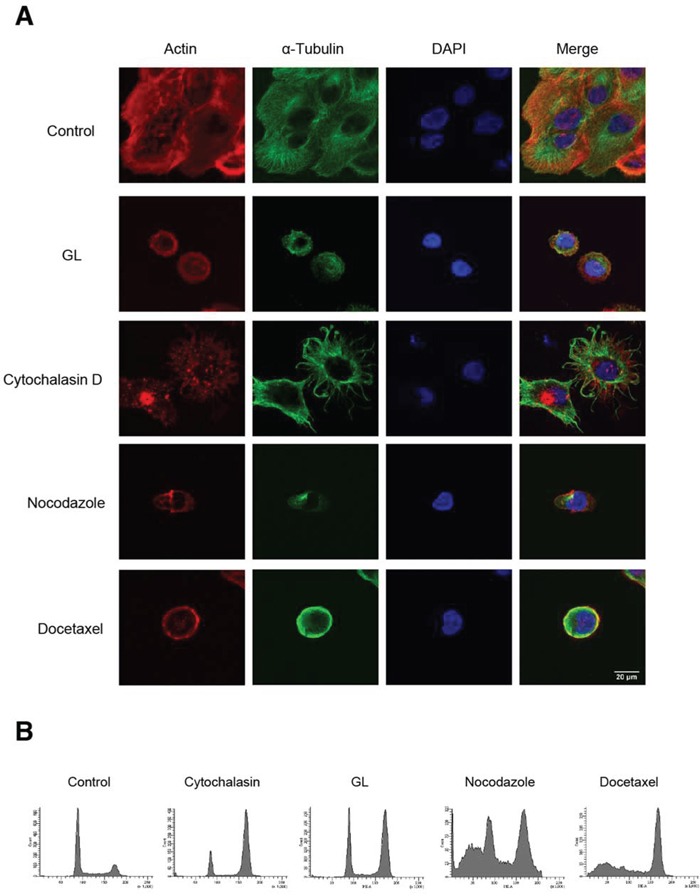
Effect of GL on cell morphology and cytoskeletal structure **A.** Double immunofluorescent staining of actin (red) and α-tubulin (green) in DU145 cells treated with cytochalasin D (10 μM), GL (10 μM), nocodazole (100 ng/ml) and docetaxel (10 nM) for 6 h. The nuclei were counterstained with DAPI (blue). Cells were visualized by confocal microscopy (x63). **B.** Representative cell cycle profiles obtained by FACS at 24 h after the treatment with the indicated compounds.

We found that after 6 h GL produces a change in morphology, clearly reducing cell size to that observed in DU145 cells arrested in mitosis. Also, GL treatment does not cause aggregation of actin as observed after cytochalasin D treatment. However, GL was able to produce a similar microtubule destabilization observed with microtubule-targeting agents (MTAs) docetaxel and nocodazole (Figure 3A). MTAs but not GL induced an increase in the percentage of subdiploid cells (sub G_0_/G_1_) that corresponds to apoptotic cells after 24 h treatment, indicating that the action mechanism of MTAs and GL should be different (Figure 3B). Accordingly, subdiploid cells in GL-treated DU145 cells are detected only after 48 h treatment (data not shown).

**Figure 4:**
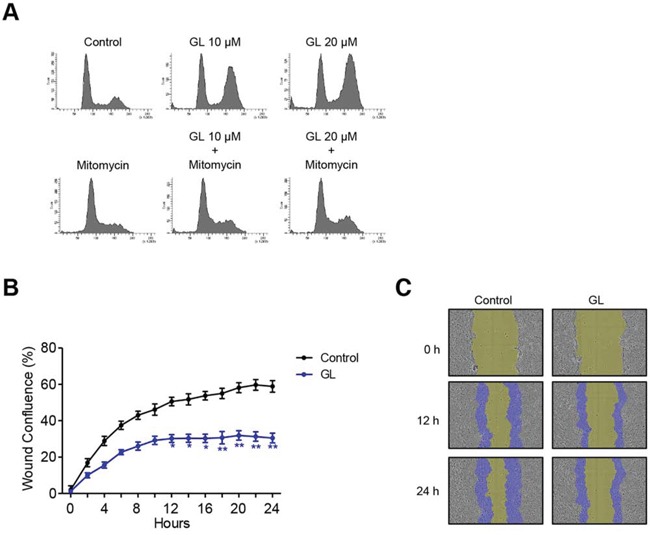
GL inhibits cell motility **A.** DU145 cells were pre-incubated with mitomycin C (5 μg/ml) for 1 h and treated with GL at 10 and 20 μM for 24 h and cell cycle analyzed by PI staining and flow cytometry. Representative histograms are shown. **B.** DU145 cells were pre-incubated with mitomycin C (5 μg/ml) for 1 h, treated or not with GL at 10 μM for 24 h and relative wound density analyzed at different time points over a period of 24 h. The measurements are from wounds made on a monolayer of DU145 cells cultured in the presence of GL and control. Data are the means of three experiments ± SE. *P<0.05; **P<0.01 compared with the control group. **C.** Images of wound healing assay were obtained at 0, 12 or 24 h and the blue areas show the initial wound boundaries at 0 h.

In order to evaluate if GL causes cell cycle arrest through *de novo* protein and RNA synthesis we used the transcriptional inhibitor mitomycin C. In the combined treatment we observed that cell cycle arrest produced by GL at 24 h was reversed with mitomycin C in DU145 cells, indicating that cell cycle arrest at G_2_/M produced by GL requires *de novo* transcription of genes involved in cell cycle checkpoints regulation (Figure 4A). Recently, it has been shown that GL inhibits invasion in DU145 cells [22]. This finding, together with the effect on microtubules stabilization shown above, has led us to investigate the effects of GL on migration process by wound healing assay. We found that GL clearly impaired wound healing in DU145 cells compared to untreated cells (Figures 4B and 4C).

### GL activates ATM/ATR signaling pathway without induce massive DNA damage

**Figure 5:**
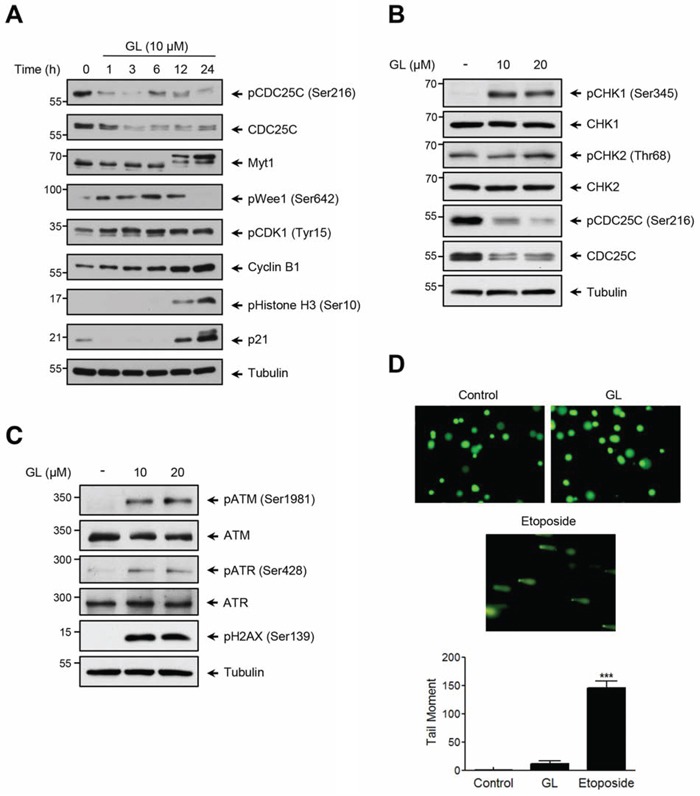
Effect of GL on the expression of cell cycle proteins and DNA damage **A.** Kinetic analysis on the steady state of proteins involved in G_2_/M phase. DU145 cells were treated with GL (10 μM) for the indicated times and the expression of the different proteins analyzed by western blots. **B.** Protein expression of pCHK1, pCHK2 and CDC25C and **C.** pATR, pATM, and γH2AX was evaluated by immunoblot in cells stimulated with GL for 24 h. **D.** Alkaline comet assay was performed to determine DNA fragments in DU145 cells treated with either GL (10 μM) or etoposide for 24 h. Representative images of alkaline comet assay and a graph with the tail moment are shown. ***P<0.001 compared with the control group.

To examine the molecular basis by which GL induces G_2_/M cell cycle arrest we firstly analyzed the expression of key proteins involved in cycle progression and checkpoint response. DU145 cells were stimulated with GL and the expression kinetic of the indicated proteins was analyzed. As shown in Figure 5A, the protein levels of pCDC25C (Ser216), CDC25C and pWee1 (Ser642) were clearly down-regulated in a time-dependent manner in response to GL treatment. By contrast, other proteins such as Cyclin B1, pHistone H3 (Ser10) or p21 were up-regulated. No significant change was observed in pCDK1 (Tyr15) and Myt1 expression levels, while Myt1 hyperphosphorylation was clearly detected after 12 h of treatment. In summary, these results clearly indicate that GL may induce cell cycle arrest through the control of the expression of key proteins involved in the regulation of S and G_2_/M phases.

CDC25C is an essential protein for the control of the G_2_/M cell cycle transition, and also a key component of the checkpoint pathways that become activated in response to DNA damage or environmental insults. Under this stress situation, ATM and ATR kinases and their downstream checkpoint kinases CHK1 and CHK2, mediate the inhibition and/or degradation of CDC25C. Based on the ability of GL to mediate CDC25C degradation, we decided to analyze whether GL may activate the ATM/ATR pathway. To study this possibility, we first monitored CHK1 and CHK2 activation levels by analyzing the phosphorylation at Ser345 and Thr68, respectively. We show in Figure 5B that GL treatment led to CHK1 phosphorylation in a dose-dependent manner, not affecting the phosphorylation levels of CHK2. CHK1 activation correlated with phosphorylation of CDC25C at the Ser216 site and posterior degradation. Similar results were obtained in PC3 cells (Supplementary Figure 2). These results indicate that GL-mediated down-regulation of CDC25C paralleled with CHK1 activation.

Next, to examine the capacity of GL to induce activation of DNA damage sensor kinases ATM/ATR, DU145 cells were stimulated with GL and ATM Ser1981 and ATR Ser428 phosphorylation detected by immunoblotting. In parallel, we evaluated Ser139 phosphorylation of histone H2A variant H2AX as marker of DNA damage. As shown in Figure 5C, GL induced ATM and ATR phosphorylation in a dose-dependent manner, affecting Ser139 phosphorylation levels of H2AX, with similar results were found in PC3 cells (Supplementary Figure 2). Finally, we performed a Comet-assay to determine DNA strand breaks (Figure 5D). In contrast to the analysis of γH2AX, no significant changes were observed in the cells stimulated with GL. By contrast, a dramatic Comet formation was observed under etoposide stimulation. These results demonstrate that GL mediates the activation of ATM/ATR signaling pathway without DNA double strand break.

### Inhibition of ATM/ATR signaling pathway rescues GL-mediated G2/M phase cell-cycle arrest

**Figure 6:**
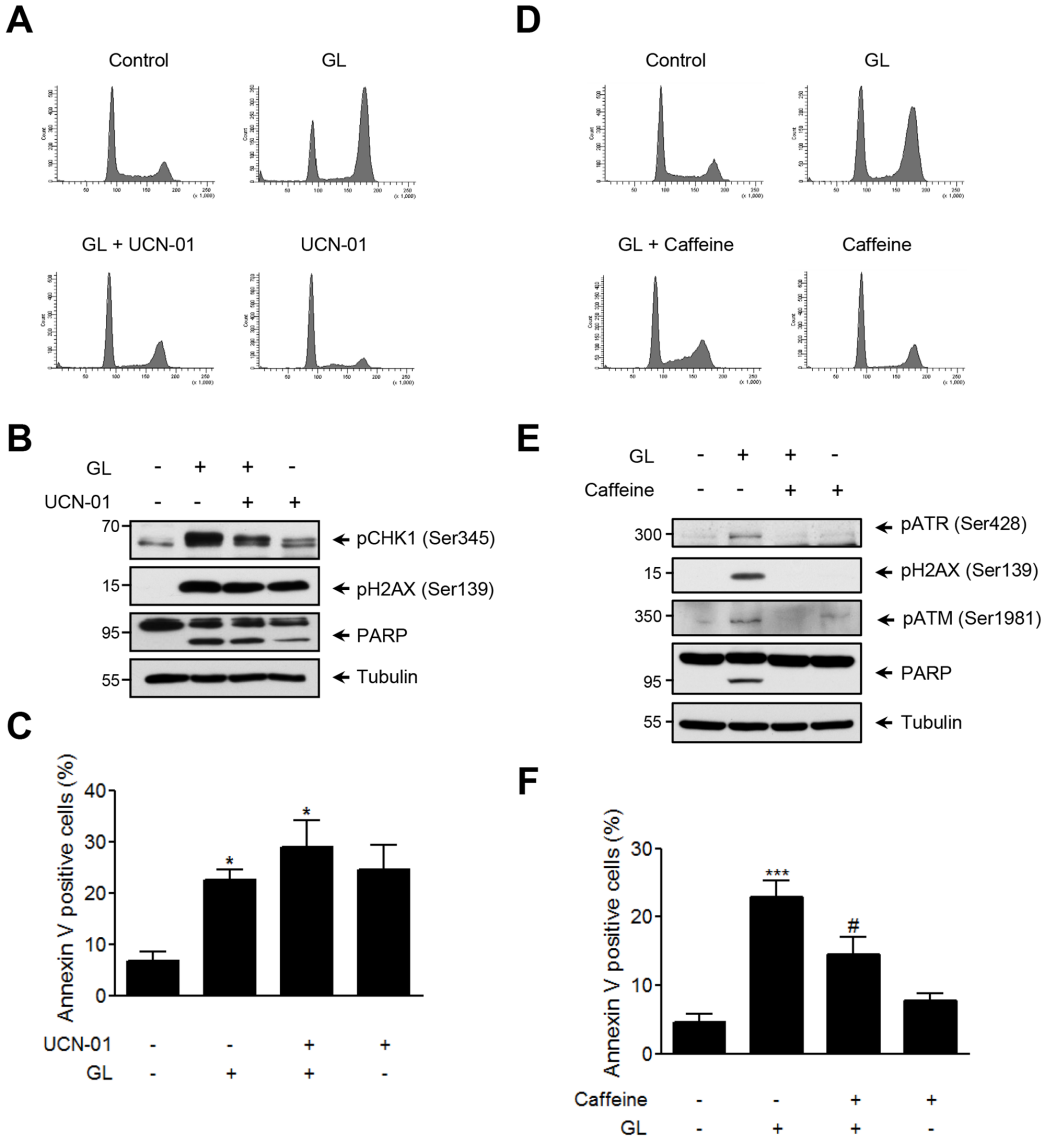
GL activates the ATM/ATR/CHK1 pathway DU145 cells were pre-incubated for 1 h with either UCN-01 (1 μM) or caffeine (10 mM) and then treated with GL 10 μM for 24 h **A, D.** Representative cell cycle profiles obtained by flow cytometry at 24 h after the treatment with the indicated compounds. **B, E.** Identification of DNA damage (pCHK1 and γH2AX) and apoptotic (PARP) proteins. **C, F.** DU145 cells were treated as above for 48 h, stained with Annexin V and PI and analyzed by FACS. Percentages of Annexin V positive cells are shown. Data are the means of three experiments ± SD. *P<0.05; ***P<0.001 compared with the control group. ^#^P< 0.05 compared with GL 10 μM group.

In view of these results, we next examined the effect of ATM/ATR inhibitors on GL-mediated G_2_/M cell cycle arrest, DDR signaling pathway and apoptosis. DU145 cells were stimulated with GL in the presence or absence of the CHK1/CHK2 dual inhibitor UCN-01, and cell cycle and the expression of pCHK1 (Ser345), γH2AX and PARP proteins evaluated in parallel. We found that inhibition of CHK1 prevented GL-mediated G_2_/M phase cell-cycle arrest (Figure 6A), but it did not interfere with GL-induced PARP cleavage (Figure 6B) and apoptosis, which was particularly increased (Figure 6C). Finally, and to further verify the role of ATM/ATR in GL-mediated G_2_/M cell cycle arrest, we performed similar experiments using the ATM/ATR inhibitor caffeine. DU145 cells stimulated with GL, in the absence or presence of caffeine, showed that ATM/ATR inhibition clearly rescued GL-mediated G_2_/M cell cycle arrest (Figure 6D), and prevented ATR, ATM and H2AX activation (Figure 6E). In contrast to the results obtained with CHK1/CHK2 inhibition, caffeine produced a significant reduction in GL-induced PARP cleavage and apoptosis (Figure 6F). Similarly, caffeine stimulation reverted GL capacity to impair wound healing in DU145 cells (Supplementary Figure 3). Altogether these data demonstrate that GL-mediated G_2_/M cell cycle arrest is mediated by activation of the ATM/ATR signaling pathway.

### N-acetyl cysteine (NAC) suppresses cell cycle arrest and apoptosis produced by GL

**Figure 7:**
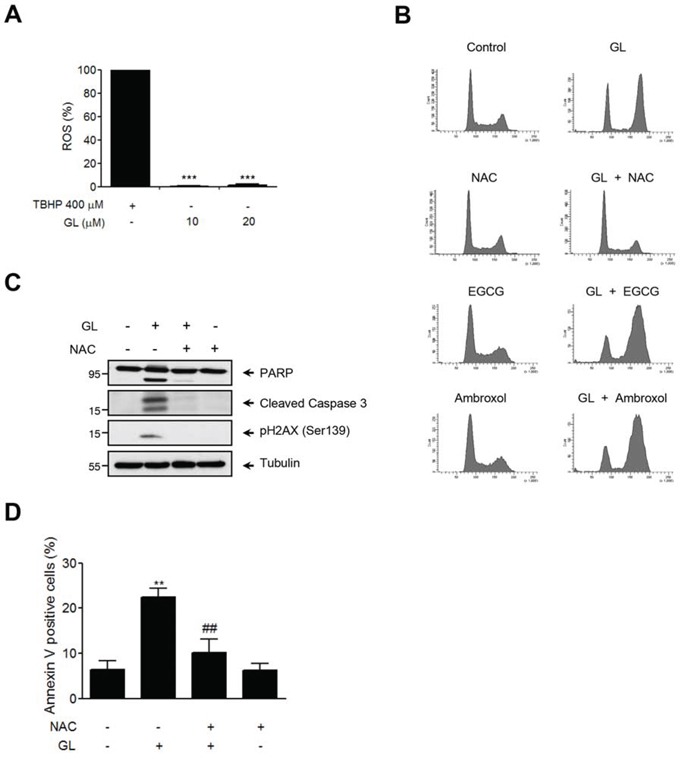
NAC inhibits GL-induced cell cycle arrest and apoptosis in DU145 cells **A.** DU145 cells were treated with either GL or TBHP and the generation of intracellular ROS was determined with fluorescence probe DCFH_2_-DA. ***P<0.001 compared with the positive control group. **B.** DU145 cells were pre-incubated either NAC (1 mM), epigallocatechin (100 μM) or ambroxol (100 μM) followed by GL 10 μM treatment. Representative cell cycle profiles obtained by FACS after 24 h of treatment are shown. **C.** Protein expression of PARP, Caspase-3 and γH2AX was determined by western blot. **D.** DU145 cells were treated as above for 48 h, stained with Annexin V and PI and analyzed by FACS. Percentages of Annexin V positive cells are shown. Data are the means of triplicate experiments ± SD. **P<0.01 compared with the control group. ^##^P< 0.01 compared with GL 10 μM group.

The DDR cascade and ROS (reactive oxygen species) signaling are both involved in the induction of cell death after DNA damage. Thus, we were interested in investigating whether an increase of intracellular ROS was involved in GL-induction of G_2_/M cell cycle arrest and apoptosis in DU145 cells. We show in Figure 7A that, in contrast to tert-butyl hydroperoxide (TBHP), GL was not able to increase the levels of intracellular ROS. Accordingly, neither the antioxidants ambroxol nor epigallocatechin gallate (EGCG) prevented GL-induced G_2_/M cell cycle arrest (Figure 7B). Interestingly, N-acetyl cysteine (NAC) treatment prevented the effect of GL on G_2_/M cell cycle arrest (Figure 7B), PARP cleavage, H2AX phosphorylation (Figure 7C) and apoptosis (Figure 7D). NAC is a scavenger of oxygen free radicals and a precursor of L-cysteine. GL has the ability to modify and covalently bind to cysteines, at least in the STAT3 protein, and therefore it is possible that NAC could bind GL attenuating its apoptotic effects.

### *In vivo* effect of GL on H2AX phosphorylation in cancer prostate

**Figure 8:**
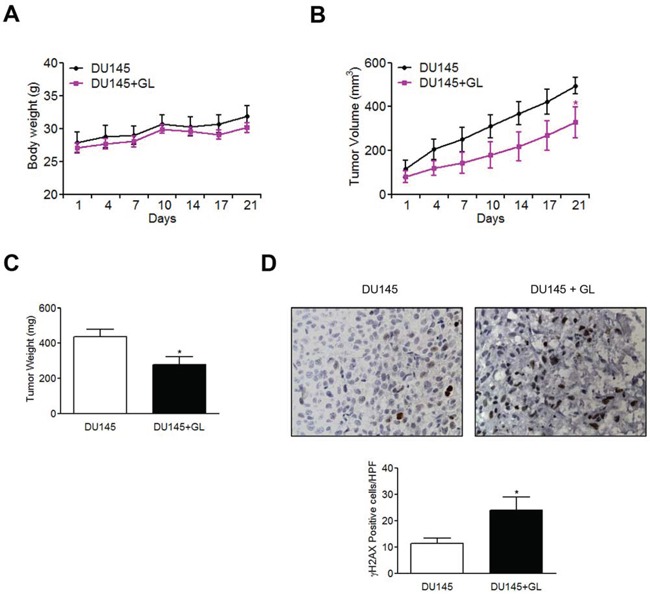
GL induces H2AX phosphorylation in prostate tumors Athymic nude-Foxn1^nu^ mice were injected subcutaneously with DU145 cells. After 4 weeks tumors were established and mice were treated every day during 21 days with GL (3 mg/kg) or DMSO (0.1%). **A.** Body weight in DU145 and GL-treated groups. **B.** Tumor volume (mm^3^) was evaluated every 3-4 days using a caliper. **C.** Tumor weight. **D.** γH2AX expression in tumor sections of controls and GL-treated mice DU145. Representative images are shown (x40). Quantitation of γH2AX positive cells/HPF (high power field) as shown in the graph. *P<0.05 compared with DU145 group.

Previous studies have demonstrated that GL produces a decrease tumor growth in several animal models of prostate cancer [20, 22]. Therefore, next we were interested in studying DDR after GL treatment *in vivo*. DU145 cell xenograft mouse model received a dose of 3 mg/kg through i.p injections every day for 21 days. Our results demonstrated that GL did not affect body weight of mice (Figure 8A). By contrast, a significant reduction of the volume tumor was observed during the treatment (Figure 8B) and the tumor weight was also significantly decreased after 21 days of GL treatment in comparison with untreated group (Figure 8C). To investigate activation of DDR signaling pathway caused by GL we determined the expression of phosphorylated H2AX. Immunohistochemistry analysis of tissue sections showed that γH2AX positive cells expression was significantly higher in mice treated with GL in comparison with untreated mice (Figure 8D). These results confirm that activation of DNA damage signaling occurs *in vitro* and also *in vivo*.

## DISCUSSION

STAT3 and NF-κB have been identified to be involved in the processes of cell proliferation, cell differentiation and cell survival and, therefore, play an important role in tumorigenesis. In addition, it has been described that constitutive activation of these transcription factors contributes to chemoresistance in multiple malignancies [40]. On the other hand, it has been shown that EBV infection induces STAT3 activation that suppresses the DDR by interrupting ATR-CHK1 signaling [41]. Moreover, STAT3 is required for efficient repair of damaged DNA following UVB irradiation and STAT3 deficient cells have reduced activity of ATM-CHK1 pathway [42]. Also, it has been well-established that cytotoxic drugs and ionizing radiation activate NF-κB [40] involved in DNA repair mechanisms [43]. Therefore, NF-κB inhibitors administered in combination with cytostatic drugs enhanced the cytotoxicity activities of these treatments favoring pro-apoptotic cascade [44].

**Figure 9:**
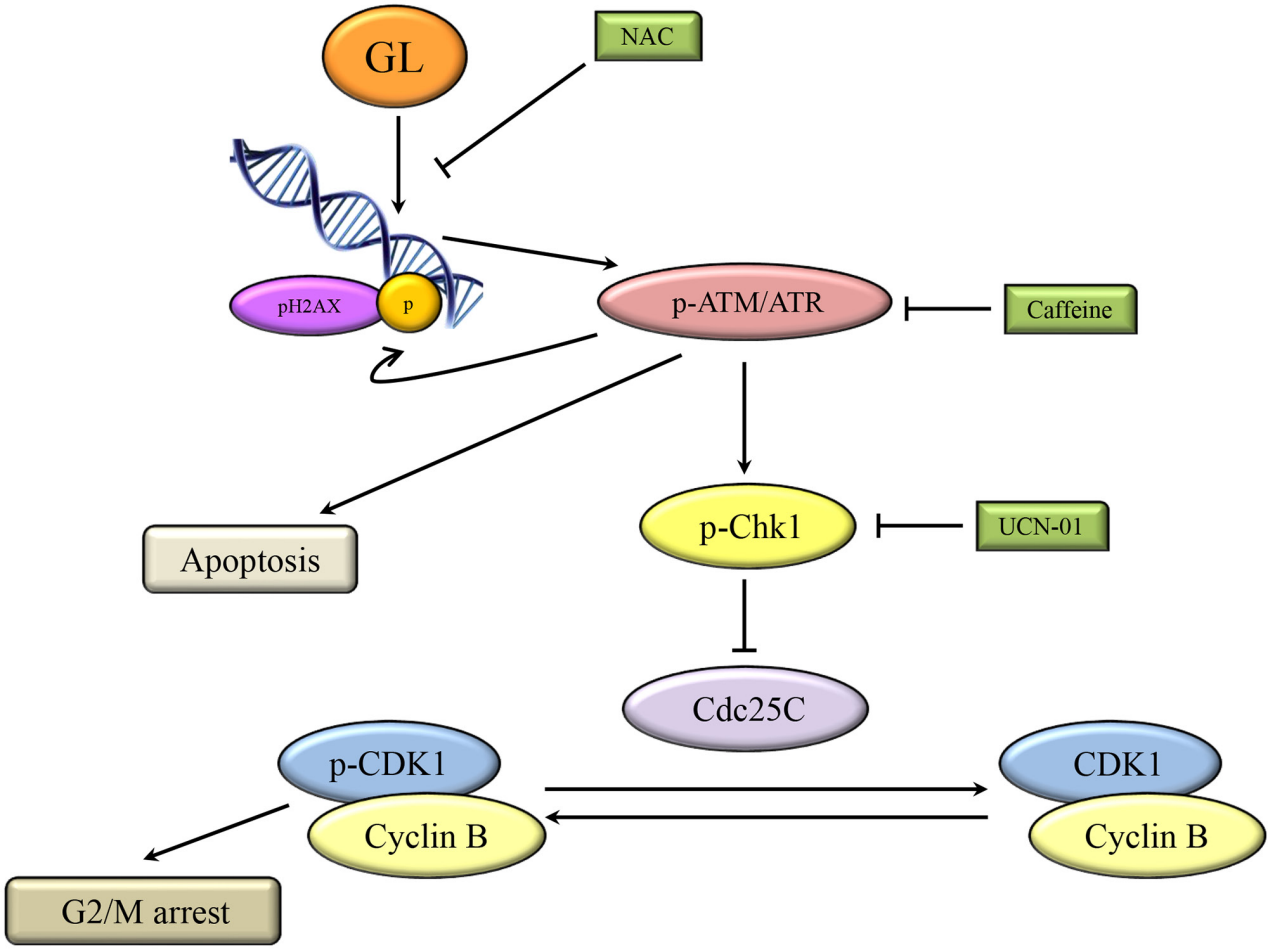
Schematic model for the ability of GL to induce cell cycle arrest and apoptosis GL induces DNA damage and activation of pATM/ATR, which phosphorylates H2AX and CHK1 to induce cell cycle arrest at the G_2_/M phase by CDC25C inhibition. Activation of pATM/ATR also induces apoptosis through a CHK1-independent pathway.

It has been previously shown that GL is a dual NF-κB /STAT3 inhibitor, but nothing is known about its effects on cell cycle and DDR signaling in cancer cells. In this study, our results demonstrate that GL was able to induce cell cycle arrest at G_2_/M phase in human prostate cancer cell lines (DU145 and PC3), with similar results in other cancer cell lines like Jurkat and SK-N-SH (data not shown). Similarly, GL induces apoptosis in androgen-insensitive prostate cancer cells through activation of ATM/ATR-CHK1 signaling without inducing DNA break. Thus, GL may exert antitumoral activity at different levels: inhibiting the action of the pro-survival transcription factors STAT3/NF-κB, inducing DNA damage signaling pathway and inhibiting DNA repairing mechanism (Figure 9). However, further studies are required to confirm that G_2_/M cell cycle arrest and activation of ATM/ATR-signaling depend on these transcription factors.

Previous studies have shown that GL produces caspase-3 dependent apoptosis in prostate cancer cells [20, 21]. Treatment with Z-VAD-FMK, a pan caspase inhibitor, prevented apoptosis, corroborating previous results, although cell cycle arrest was maintained at G_2_/M in DU145 cells. These results demonstrate that a repair process is activated after GL in spite of blocking apoptosis cell death, showing that they are processes with different signaling pathways.

In some cases, such as after treatment with docetaxel or cytochalasin D, cell cycle arrest is accompanied by cytoskeleton disorganization at actin/tubulin levels [45–47]. Microtubule-targeting agents (MTAs) arrest mitosis and in turn cell death. Recently, an study have proposed that, instead of mitotic arrest, disrupting intracellular trafficking of DNA repair proteins (ATM, ATR, DNA-PK, Rad50, Mre11, p95/NBS1, p53, 53BP1 and p63) is responsible of MTAs action mechanisms, such as vincristine or paclitaxel. Also, the treatment with these MTAs results in higher and sustained γ H2AX levels in A549 and MCF7 cells treated with radiation or etoposide. For this reason, the combination of MTAs plus DNA damaging agents enhances their effect separately [48]. Our results show that GL treatment provokes microtubule alteration at early stages in DU145 cells and thus might be hindering the transport of proteins involved in repairing the process, promoting cell arrest in G_2_/M and apoptosis.

Besides the ability to inhibit NF-κB/STAT3, we show that GL may target cancer cell progression by the activation of ATM/ATR pathway, inducing a rapid CDC25C degradation in DU145 cells. CDC25C protein stability is regulated by DDR kinases ATM/ATR-CHK1/CHK2. We identified that GL activates DDR in DU145 cells through ATR and ATM phosphorylation, which increases CHK1 phosphorylation at Ser345 but not phosphorylation of CHK2 at Thr68. Depending on the cellular context and DNA damage agents, ATM and ATR or both kinases may be activated. For example, whereas VP-16 provokes ATR activation [49], camptothecin activates either ATM or ATR in DNA damage events in different cancer cell lines [50]. The critical role of ATM/ATR-CHK1 in the regulation of GL-mediated degradation of CDC25C was confirmed by the inhibition of the ATM/ATR-CHK1 checkpoint control pathway using caffeine and the CHK1 inhibitor UCN-01.

ATM/ATR pathway is activated mainly in response to different types of DNA damage. Although we observed an increase in γH2AX levels in GL treated DU145 cells and animal models, GL did not induce massive DNA DSBs as compared with etoposide in comet assays. One possibility is that GL could be causing another type of DNA damage similar to that caused by alkylating agents, transferring methyl or ethyl group to a DNA base. The capacity of GL to covalently bind to cysteine could block the active cysteine site of O^6^-methylguanine methyltransferase (MGMT), inhibiting this protein and therefore preventing this DNA repair mechanism, activating DNA response signaling. DNA alkylating agents such as temozolomide, carmustine and estramustine are used in cancer therapy [51]. On the other hand, it has been reported that oxidative stress can also activate ATM by a mechanism independent of DNA DSBs [52]. In this sense, we show that GL does not produce ROS in DU145 cells but their effects on cell cycle and apoptosis were blocked with NAC treatment. NAC is an aminothiol used as an antioxidant, but it is also a synthetic precursor of GSH and intracellular cysteine, and can interact with proteins presenting cysteine residues or thiol groups [53, 54]. In this context, a previous study showed that GL is a cysteine reactive inhibitor that covalently binds to cysteine in STAT3 inhibiting its binding to DNA. Therefore, while NAC could block the reactive site of GL, we cannot exclude oxidative stress to be involved in GL-mediated activation of ATM/ATR pathway.

In summary, this study reports for the first time a new mechanism of action for GL, with important consequences on the damage response pathway in the context of its antitumor activities on prostate cancer. We demonstrate how fungal GL induces DDR via ATM/ATR-CHK1 pathway, causing apoptosis and G2/M cell cycle arrest in prostate cancer (Figure 9), inhibiting tumor growth *in vivo* in mouse tumor models. These findings validate GL as a promising antitumor agent for treatment of castration-resistant prostate cancer, alone or in combination with other drugs.

## MATERIALS AND METHODS

### Cell culture and reagents

DU145, PC3 and RWPE-1 cells were from the American Type Culture Collection (ATCC, Manassas, VA, USA). DU145 and PC3 cells were cultured in RPMI 1640 medium supplemented with 10% of fetal bovine serum (FBS) and 1% penicillin/streptomycin. RWPE-1 cells were cultured in keratinocyte serum free medium supplemented with bovine pituitary extract (0.05 mg/ml) and human recombinant epidermal growth factor (5 ng/ml). Human normal dermal fibroblasts (HNDFs) were purchased to Innoprot (Bizkaia, Spain) and cultured in DMEM medium supplemented with 10% of FBS and antibiotics. Cells were incubated at 37°C in a humidified atmosphere containing 5% CO_2_. Cell lines were routinely tested to be free of mycoplasma and cross contamination. DU145 validation was performed by a multiplex PCR with Geneprint10 System (Promega, Madison, WI, USA) following the manufacturer's manual. Galiellalactone was isolated as previously described [55] or purchased from US Biological Life Sciences (Salem, MA, USA). Mitomycin C and Z-VAD-FMK were obtained from Enzo Life Technologies (Farmingdale, NY, USA). Antibodies against anti-phospho-ATM Ser1981 [(D6H9)-5883], anti-phospho-ATR Ser428 (2853), anti-phospho-CDC2 Tyr15 (9111), anti-Cyclin B1 (4138), anti-phospho-Histone H3 Ser10 [(D2C8)-3377], anti-Myt1 (4282), anti-phospho-WEE1 Ser642 [(D47G5)-4910], anti-p21 Waf1/Cip1 [(12D1)-2947], anti-phospho-CDC25C Ser216 (9528), anti-CDC25C [(5H9)-4688], anti-phospho-CHK2 Thr68 [(C13C1)-2197], anti-phospho-CHK1 Ser345 [(133D3)-2348], anti-PARP (9542), anti-Caspase-3 [(8G10)-9665], anti-phospho-Histone H2AX Ser139 [(20E3)-9718], anti-ATM [(D2E2)-2873], anti-ATR [(E1S3S)-13934], anti-CHK1 [(2G1D5)-2360] were purchased from Cell Signaling Technology (Danvers, MA, USA). The antibody anti-CHK2 (05-649) was obtained from Millipore (Merck Millipore, Billerica, MA, USA). The antibody anti-α-Tubulin (T9026) and anti-Flag (F3165) were obtained from Sigma-Aldrich (St Louis, MO, USA). The antibody anti-p53 was obtained from Santa Cruz Biotechnology (Santa Cruz, CA, USA). Alexa Fluor 488 was purchased from Life Technologies (Carlsbad, CA, USA). All other reagents were from Sigma Co (St Louis, MO, USA).

### FACS analysis

For cell cycle analysis, cells were fixed in 70% cold ethanol at −20°C overnight. After that cells were washed with PBS once and then stained with 1 mg/mL propidium iodide (PI) and treated with RNase A (50 U/mL) for 2 h at 37°C in darkness. For apoptosis studies, cells were harvested and washed in cold PBS and then resuspended in binding buffer consisting of 10 mM Hepes, 140 mM NaCl and 2.5 mM CaCl_2_ pH 7.4. Cells were stained with Annexin V, Alexa Fluor 488 conjugate (Molecular Probes by life technologies, Carlsbad, CA, USA) and propidium iodide. Cell cycle distribution and apoptosis were determined by BD FACSCanto™ flow cytometer (BD Biosciences, San Jose, CA, USA) using BD FACSDiva™ software.

### Western blotting

Whole cell extracts were obtained by lysing the cells in NP-40 buffer (50 mM Tris-HCl pH 7.5, 150 mM NaCl, 10% glycerol and 1% NP-40) supplemented with protease and phosphatase inhibitors. Lysate concentrations were determined by the Bradford assay (Bio-Rad Laboratories, Hercules, CA, USA). Proteins (50 μg/lane) were separated by SDS-PAGE, transferred onto PVDF membranes and blocked with PBS-T (Phosphate-buffered saline and 0.1% Tween-20) containing 5% non-fat dry milk for 1 h at room temperature (RT). Incubation with primary antibodies was performed overnight at 4°C, followed by incubation with the appropriate horseradish peroxidase-conjugated secondary antibody. The washed membranes were incubated with appropriate secondary antibodies coupled to horseradish peroxidase that were detected by an enhanced chemiluminescent reagent (ECL).

### Immunofluorescence

DU145 cells were grown on coverslips and after treatment fixed for 20 min in 4% paraformaldehyde and permeabilized in 0.12% Triton X-100 for 10 min. After that, cells were stained for 1 h at room temperature with Phalloidin-TRITC 10 μg/mL and anti-α-Tubulin (T9026) for staining of actin filaments and microtubules, respectively, and counterstained with Alexa Fluor 488 goat-anti-mouse IgG secondary antibody. Coverslides were mounted in Vectashield mounting media with DAPI (Vector Laboratories Inc., Burlingame, CA, USA) and examined with an LSM 5 EXCITER (Carl Zeiss MicroImaging GmbH) confocal laser-scanning microscope using a 63×/1.40 oil objective (Plan-Apochromat), and ZEN 2008 software (Carl Zeiss MicroImaging GmbH).

### Measurement of ROS levels

Cells were stimulated with either GL or tert-butyl hydroperoxide (TBHP) (0.4 mM). After 3 h of incubation, cells were washed with PBS and incubated with 1 μM of 2,7-dichlorofluorescein diacetate (H2DCF-DA; Molecular Probes, OR, USA) for 20 min at 37°C in darkness. Fluorescence was measured at 450 nm excitation and 535 nm emission using a TECAN GENios Pro (Tecan Group Ltd, Switzerland).

### Wound healing assay

DU145 cells were seeded on 96-well plates (ImageLock plate, Essen Bioscience, Ann Arbor, MI, USA) at a 5 × 10^4^ cell density and were allowed to attach overnight. When cells reached confluence, a wound was scratched across each well using Wound Maker device (Essen Bioscience) and detached cells were removed by gentle washing with PBS. Then, cells were treated or not with GL in the presence of mitomycin C (5 μg/ml). Images have a blue mask showing the initial wound boundaries at 0 h and wound closure was monitored hourly for 24 h and quantified as wound confluence (%) with IncuCyte ZOOM Live-Cell Imaging System (Essen Bioscience, Hertfordshire, UK).

### Comet assay

Cells (4 × 10^5^) were seeded into 6-well plates and treated with GL or etoposide for 24 h. DNA damage was detected using an OxiSelect™ Comet Assay kit (Cell Biolabs Inc, San Diego, USA) following the manufacturer's instructions. Briefly, cells were harvested and mixed with low melt agarose on the OxiSelect Comet Slide. Then, the embedded cells were lysed and treated with alkaline solution to denature DNA. After that, electrophoresis was carried out under alkaline conditions at 1 V/cm and 300 mA for 30 min and the samples stained with Vista Green fluorescence dye for 15 min in darkness, analysed using a Leica DM2500 fluorescent microscope and quantified by Casp software (CASPlab, Wroclaw, Poland).

### Prostate tumor xenograft model

Six-week-old male athymic nude-Foxn1nu mice (Harlan Laboratories, Barcelona, Spain) were kept on a 12 h light-dark cycle, temperature 20°C (±2°C) and 40-50% relative humidity with free access to standard food and water. Experimental procedure was performed in accordance with European Union and the Córdoba University ethical committee guidelines (2014PI/015). A total of 10 mice were injected subcutaneously into the flank with 3×10^6^ DU145 cells and tumors were established 4 weeks after injection. Then, mice were divided into 2 groups: Group 1 (n=5) received vehicle (1% DMSO in PBS) and group 2 (n=5) were treated with 3 mg/kg of GL by intraperitoneal injection (i.p) every day for 3 weeks in both cases. Tumor volume was measured every 3-4 days using a caliper and calculated by the formula length (mm) x width x height x 0.5632. After treatment, mice were sacrificed and tumours were extracted, weighted, fixed in 4% paraformaldehyde and then embedded in paraffin. Sections (5 μM thickness) of each tumour were prepared for immunohistochemistry analysis.

### Immunohistochemistry

The sections of xenograft tumor samples were deparaffinized in xylene and rehydrated through a graded ethanol series ending in water. Antigen retrieval was done by heating in sodium citrate 10 mM pH 6 at 98°C for 10 min and then incubated in methanol and 0.3% hydrogen peroxide for 30 min to block endogenous peroxidase activity. Non-specific binding was blocked with IHC Select Blocking Reagent (Merck Millipore, Billerica, MA, USA) at room temperature for 30 min followed by incubation with the phospho-histone H2AX (Ser139) primary antibody at 4°C overnight. After that, samples were incubated with secondary antibody goat anti-rabbit (Merck Millipore, Billerica, MA, USA) at room temperature for 4 h. Incubation with the IHC Select Streptavidin-HRP reagent was performed at room temperature for 30 min, before the chromogen was developed using diaminobenzidine according to the manufacturer's instructions (Merck Millipore, Billerica, MA, USA). A Leica DM2500 microscope and a Leica DFC420c camera were used for slide observation and photography and all image processing was done using ImageJ (Bethesda. MD, USA).

### Statistical analysis

Data are expressed as mean ± SD, except for wound healing assay, whose data are represented as mean ± SE. Animal studies are expressed as mean ± SEM. Differences were analyzed by one-way ANOVA test followed by Newman-Keuls post hoc test. P< 0.05 was considered statistically significant. Statistical analysis was performed using GraphPad Prism version 5.00 (GraphPad, San Diego, CA, USA)

## SUPPLEMENTARY FIGURES


