# Supplementary material for: Galiellalactone induces cell cycle arrest and apoptosis through the ATM/ATR pathway in prostate cancer cells
Source: Oncotarget. 2015 Dec 14;7(4):4490–506. doi: 10.18632/oncotarget.6606 (PMC4826221; doi:10.18632/oncotarget.6606)
Supplement: Supplementary file 1 [file oncotarget-07-4490-s001.pdf]

SUPPLEMENTARY FIGURES

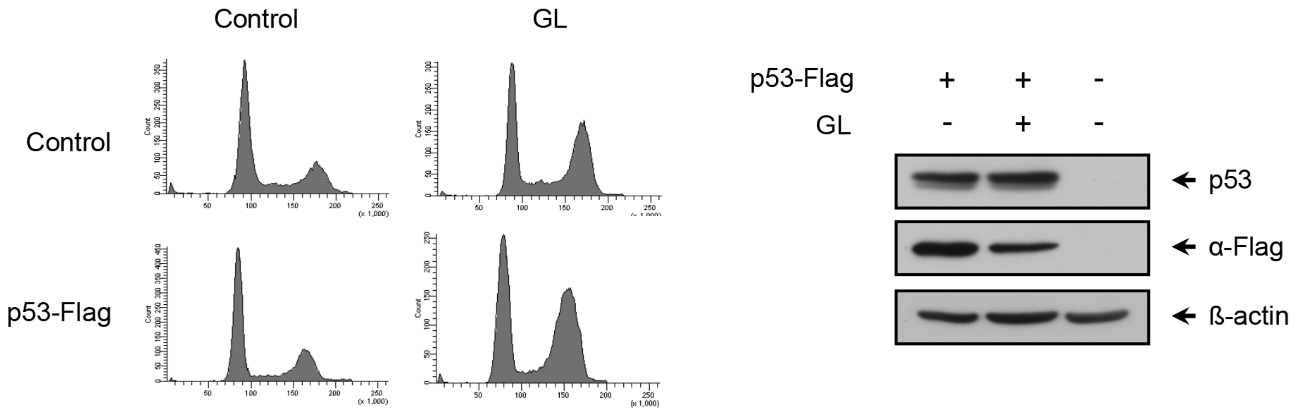

Supplementary Figure S1: GL induces G2/M phase cell cycle arrest independent of p53. PC3 cells were transfected with p53-Flag as indicated and after 24 hours stimulated with GL (10 µM) for another 24 hours. A fraction of the cells was used for cell cycle analysis (left panel) using flow cytometry. Representative histograms are shown. Right panel shows protein expression analyzed by immunoblot.

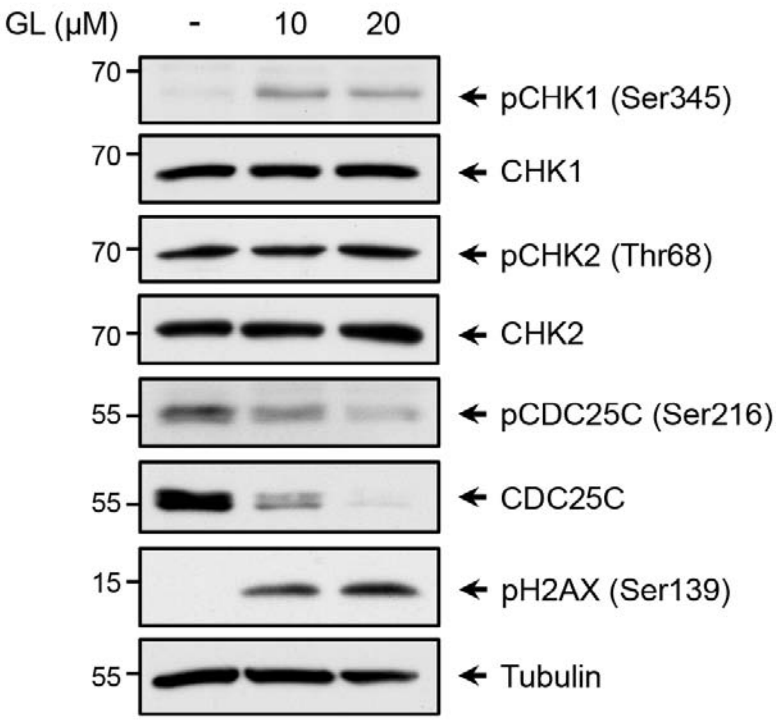

Supplementary Figure S2: Effect of GL on the expression of cell cycle and DNA damage proteins in PC3 cells. PC3 cells were treated with GL as indicated for 24 hours, lysed and protein expression analyzed by immunoblot.

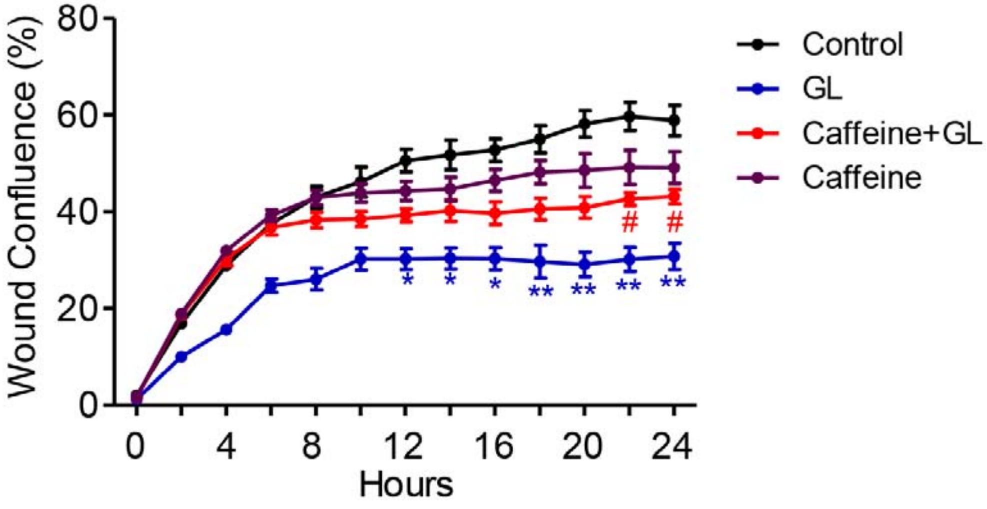

Supplementary Figure S3: Caffeine stimulation reverts GL capacity to impair wound healing in DU145 cells. DU145 cells were pre-incubated with mitomycin C (5 µg/ml) for 1 h, in the absence or presence of caffeine (10 mM), stimulated with GL (10 µM) and relative wound density analyzed at different time points over a period of 24 h. Measurements are from wounds made on a monolayer of DU145 cells cultured under the indicated conditions. Data are the means of three experiments ± SE. *P<0.05; **P<0.01 compared with the control group. #P< 0.05 compared with GL group.
